# Development of a quality of life measure for left ventricular assist device recipients using a mixed methods approach

**DOI:** 10.1002/ehf2.14850

**Published:** 2024-06-14

**Authors:** Anita L. Slade, Christel McMullan, M. Sayeed Haque, Stephen Griffith, Laura Marley, David Quinn, Margaret E. O'Hara, Mike Horton, Melanie J. Calvert, Hoong Sern Lim, Deirdre A. Lane

**Affiliations:** ^1^ Centre for Patient Reported Outcomes Research, Institute of Applied Health Research University of Birmingham Birmingham UK; ^2^ National Institute for Health and Care Research Birmingham Biomedical Research Centre University of Birmingham Birmingham UK; ^3^ National Institute for Health and Care Research Blood and Transplant Research Unit in Precision and Cellular Therapeutics University of Birmingham Birmingham UK; ^4^ Institute of Applied Health Research University of Birmingham Birmingham UK; ^5^ University Hospital Birmingham NHS Foundation Trust Birmingham UK; ^6^ Academic Department of Rehabilitation Medicine, Leeds Institute of Rheumatic and Musculoskeletal Medicine University of Leeds Leeds UK; ^7^ Birmingham Health Partners Centre for Regulatory Science and Innovation University of Birmingham Birmingham UK; ^8^ National Institute for Health and Care Research (NIHR) Applied Research Collaboration West Midlands Birmingham UK; ^9^ Department of Cardiovascular and Metabolic Medicine and Liverpool Centre for Cardiovascular Science University of Liverpool Liverpool UK; ^10^ Department of Clinical Medicine Aalborg University Aalborg Denmark

**Keywords:** health‐related quality of life, heart failure, left ventricular assist device, patient‐reported outcomes, Rasch analysis

## Abstract

**Background:**

Left ventricular assist device (LVAD) recipients report symptom improvement but find adjusting to life with the LVAD challenging. These challenges are unique, and existing patient‐reported outcome measures (PROMs) do not reflect their experiences. This study aimed to develop a culturally relevant quality of life PROM for use with LVAD recipients in future research, design evolutions and clinical practice.

**Methods:**

A three‐stage mixed‐methods approach was used to develop a PROM: stage 1 included group concept mapping (GCM); stage 2 semi‐structured qualitative interviews were conducted with 11 LVAD recipients and 10 clinicians, and a questionnaire was developed using a conceptual framework; and stage 3 used exploratory psychometric analysis of the PROM data using Rasch measurement theory. This paper presents stages 2 and 3.

**Results:**

The conceptual framework consisted of four key concepts, including general health, life with the LVAD, equipment and clothing and emotional impact. Statements from interviews and GCM were used to create items for the LVAD quality of life (LVAD‐QoL). Cognitive interviews tested face validity and participant comprehension. Forty‐nine participants were recruited from three UK transplant centres. PROM data were collected and analysed using Rasch analysis. Four items displayed misfit; dependency between item sets was the biggest issue (57/485 pairwise differences). After restructuring and dealing with item misfit, the LVAD‐QoL conformed to the Rasch model, supporting the psychometric properties and quality of the LVAD‐QoL.

**Conclusions:**

Using a mixed‐methods approach ensured the development of a robust and psychometrically sound tool for research, design evolution and clinical practice with LVAD recipients.

## Background

Heart failure affects approximately 26 million people.^1^ Mortality rates from advanced heart failure currently stand at 50% within 1 year.^2^, ^3^ When therapeutic options are exhausted, access to a donor transplant is needed, but this is often limited by donor shortages. Implanting a left ventricular assist device (LVAD) is an alternative option, but accessibility varies by country, and in the UK, they are only available as a bridge to transplant. Barring complications, recipients join a low priority list for future transplantation; consequently, many recipients live with the LVAD long term. Improvements in LVAD design and surgical procedures have improved life expectancy. While recipients experience improvement in symptoms, LVAD implantation can bring additional complications, including driveline infections, blood clots, stroke and anaemia.^4^, ^5^ The post‐implant journey requires constant monitoring, and studies have shown that the psychological and physical challenges can be difficult for the recipient and their family.^5^, ^6^, ^7^

Patient‐reported outcome measures (PROMs) can be used to monitor the impact of the LVAD on health‐related quality of life.^7^, ^8^ Using PROMs in clinical practice can improve utilisation of clinical resources, survival rates, patient satisfaction with symptom management and clinical consultations, as well as identifying potential adverse events.^9^, ^10^ PROMs can play an important role in design evolutions and clinical trials by providing data on the tolerability, efficacy and safety of medical devices.^7^, ^11^, ^12^ However, this does require PROMs to be comprehensive and capture domains that matter to LVAD recipients.^4^, ^5^, ^13^ Research has shown that PROMs are more sensitive when recipients with lived experience of the condition have contributed to their development.^14^

Discussions with our LVAD patient and public involvement (PPI) group found that many of the current cardiology‐specific PROMs no longer applied to them, and they experienced unique challenges not captured by generic measures. The group identified a wide range of issues that they felt required a specific PROM to reflect their unique experiences. Patrick *et al*. (2011) recommend that the culture and language of the population of interest be an important consideration when developing a new PROM. This may be reflected in differences in measurement concepts, standards of treatment and disease progression.^14^ A recently published quality of life measure did not identify some of the issues that our participants identified as important for inclusion in a PROM, and an emphasis on faith was not considered appropriate by our participants.^15^, ^16^ Consequently, there is not a culturally appropriate LVAD PROM that can be used to monitor the impact of receiving an LVAD on health‐related quality of life in the UK. This paper presents a mixed‐methods approach to the development of a health‐related quality of life PROM for use with LVAD recipients in clinical practice and research.

## Methods

Ethical approval was received from the NHS Health Research Authority (Ref. No. 19/WM/0120) and complies with the Declaration of Helsinki. The development of the PROM consisted of three stages, including (1) group concept mapping (GCM), (2) qualitative interviews and (3) psychometric analysis. Patients over 18 with experience of living with an LVAD were eligible for inclusion. Patients with insufficient English to complete the PROM, severe communication problems, terminal illness, severe mental health problems or severe cognitive impairment were excluded.

### Stage 1 group concept mapping

GCM is a semi‐quantitative mixed‐methods approach that identifies and structures recipients' experiences of living with an LVAD. Data were collected and analysed using groupwisdom™ concept mapping software and consisted of three stages.^17^ Stage 1 participants generate statements in relation to the prompt ‘Life with an LVAD means …’. In stage 2, participants reviewed the final generated list of statements and arranged them into perceived thematic groups, which they characterised using their own labels according to content. In stage 3, participants rated statements according to their perceived relevance, frequency of experience and importance for inclusion in a PROM. Cluster analysis defined the relationships and commonalities between all the participants' responses and statement priorities. These were used to develop a conceptual framework and identify the range of issues recipients considered important for inclusion in an LVAD‐specific PROM.^5^, ^17^, ^18^ Full details of the GCM have been published elsewhere.^5^

### Stage 2 qualitative interviews

Qualitative interviews explored recipients' experiences of living with the LVAD and its impact on their families and health‐related quality of life. A topic guide was developed using the results from the GCM. Semi‐structured interviews were carried out with current and previous LVAD recipients and clinicians working in this area.^19^, ^20^ The topic guide was flexible and evolved as suggested by emerging findings.

Participants for the interviews were recruited from a single UK heart transplant centre. Potential participants were contacted by LVAD co‐ordinators and given a consent‐to‐contact form. Those who agreed to be contacted were sent the participant information sheet, consent form and demographic questionnaire. Prior to COVID‐19, participants were given the option of in‐person or telephone interviews. During COVID‐19 restrictions, participants were offered telephone or online Zoom interviews, which were audio‐recorded and transcribed by a professional transcription company. On completion of the interviews, participants were asked if they would be willing to take part in cognitive interviews and review a draft of the new PROM. A cross‐section of staff, including nurses, psychologists, technicians and clinicians, was recruited using known contacts and snowballing. That is, staff known to the research team were asked to send invitation letters and consent‐to‐contact forms to colleagues working with LVAD recipients. Staff who consented to contact were sent an information sheet, a consent form and a demographic questionnaire. COVID‐19 restrictions meant that staff interviews were conducted individually online, audio‐recorded and transcribed. Interviews were conducted by A. L. S. using the same topic headings as used with LVAD recipients.

Subsets of transcripts were coded separately by two researchers (A. L. S. and C. M.) to ensure consistency of coding methods and the coding guide. Disagreements would be moderated by M. J. C. if necessary. Following initial familiarisation with the data, transcripts were analysed thematically using line‐by‐line descriptive, inductive coding and NVivo software, with the aim of exploring and developing the main themes in the data.^21^ Codes and quotations were compared across the data and inductively categorised into concepts and themes. The conceptual framework was developed based on this analysis and the GCM.

### PROM development

**FIGURE 1:**
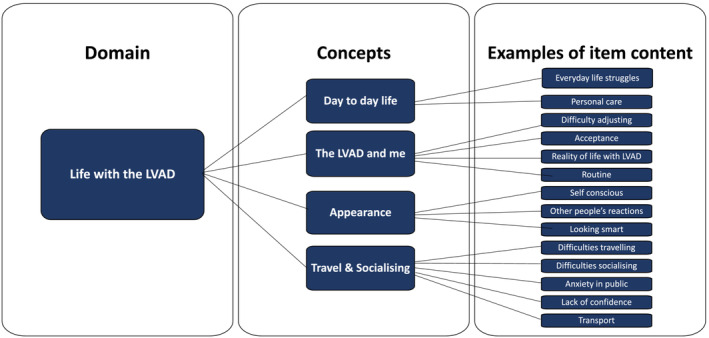
Content of life with a left ventricular assist device (LVAD) module. This figure shows one of the domains broken down into more detail with examples of item content. Statements supporting the item content were used to develop questions, and examples of some statements can be seen in *Table* 2.

Modules reflecting key concepts were used to structure a draft copy of the LVAD quality of life (LVAD‐QoL) questionnaire. Statements from the interviews and GCM that reflected concepts were utilised to develop individual questions (items). Items were selected to reflect the continuum of severity and difficulties perceived by participants. Members of the PPI group and the research team iteratively reviewed and refined the design of the LVAD‐QoL, response options, item wording and content until a draft version was ready to conduct cognitive interviews. A detailed example of one of the modules is shown in Figure 1. Examples of the types of statements used to develop items in that module can be seen in *Table* 1, and examples of quotes from life with the LVAD are shown in *Table* 2.

**Table 1:** Key concepts from qualitative interviews.

Key concepts
General health issues
Symptoms and side effects
Some recipients described improvements in their overall health and quality of life; others described ongoing residual problems and side effects, including nosebleeds and anaemia from warfarin therapies. Some participants were experiencing symptoms such as dizziness and swollen ankles. Four participants reported ongoing memory and concentration problems, which for some had resulted in the loss of employment or enforced early retirement.
Breathing difficulties and stairs
Breathlessness and lack of speed variability in the LVAD limited some activities such as exercise, mobility and climbing stairs or inclines. Shortness of breath was one of the key indicators that clinicians looked for when patients were attending clinic.
Sleep and fatigue
Half of the participants described ongoing issues with fatigue, which could be managed by pacing activities, but others were not always sure why they were feeling fatigued. Anaemia caused by warfarin therapy was a recognised cause of fatigue, and clinicians stated that it was something they regularly evaluated. Sleeping difficulties were an ongoing concern for half of the participants. Sleeping difficulties caused by the LVAD itself were highlighted by three participants; one felt that the uniform LVAD speed made falling asleep difficult as the LVAD was going too fast. Another participant described finding the internal weight of the LVAD equipment made sleeping uncomfortable, and one participant found being plugged into the mains at night limited movement and made toileting difficult. A couple of participants described the psychological impact of the LVAD surgery and described ongoing nightmares and difficult sleep patterns, which they felt were related to their time in intensive care.
Life with the LVAD
The LVAD and me
The length of time it took participants to adjust to life with an LVAD seemed to be variable, with some expressing a ‘can do’ attitude and an acceptance ‘that you had to get on with it’. A couple of participants were struggling to come to terms with the LVAD. One participant described going into a dark, horrible place when they first realised post‐operatively that the wires and machine were going to stay and the implication that this was going to be a long‐term feature of their life. One participant felt that the machine dictated their life, and they were never free from it, unlike a prosthetic limb that could be removed at the end of the day.
Day to day
Day‐to‐day routine management of the LVAD and the constraints imposed on activities of daily living and leisure were common complaints. Constantly having to be mindful of fluid intakes, maintenance of the batteries and equipment and the constraints this imposed was difficult for some participants, while others were more accepting and described it as an acceptable inconvenience considering the alternatives.
Appearance
Two participants described feeling self‐conscious about their appearance, and nearly half of the participants described difficulties in finding suitable clothing if they wanted to look smart. A couple of participants found other people's reactions to the equipment upsetting at times, and one described losing friends because of the LVAD. Clinicians and one participant discussed the particular difficulties faced by female recipients if they wanted to wear feminine clothes, such as dresses, because of the wires and access to the battery pack.
Travel and socialising
Two participants lacked the confidence to go out alone or use public transport, even pre‐COVID‐19; this was a particular concern when using crowded public transport. An issue that was raised by most of the participants related to anxiety about someone trying to snatch their equipment bag while out. Several participants described anxieties about going through airport security and being mistaken for a terrorist because they were carrying a backpack with wires showing under their clothing. Travelling with the amount of equipment required was also challenging, and participants had different strategies for dealing with it, including UK‐only vacations, going on cruises instead of flying and one participant opting to use a wheelchair at the airport.
Equipment and clothing
Equipment and clothing
All participants discussed the equipment and their maintenance routines. A universal complaint was the weight of the batteries and spare equipment, which were always required. Finding comfortable ways to carry the equipment was difficult, and many of the participants had adapted clothing or bags to suit their own needs. Finding suitable clothing was also an issue, and some participants had paid to have clothes adapted to accommodate the drivelines, etc.
Driveline and dressings
Managing the driveline and dressings was a big concern for all the participants. Fear of infection was universal, and having to rely on a family member to change the dressings because of their position was a cause of frustration for some. One participant described it as ‘an Achilles heel’ because infections could not be managed without long‐term antibiotics. Long‐standing recipients expressed concerns about the deteriorating condition of their driveline and how this would be managed in the future.
Emotional impact
Positive impact
Participants were generally positive about the LVAD and grateful for additional years and improvements in their quality of life. Participants described experiencing life events such as weddings or the birth of grandchildren, which they acknowledged would not have happened without the LVAD. However, feelings of gratitude were not unanimous; two participants expressed reservations about the LVAD, and this seemed to be related to the health issues that had led to the LVAD implant. Both described being fit and well prior to needing the LVAD, and consequently, they had found it difficult to adapt to its restrictions. Other participants, with previously poor health and a limited quality of life, felt the LVAD had improved their lives. Staff also acknowledged that prior health conditions could influence post‐operative acceptance of the LVAD.
Relationships and family
While recipients were relieved to be alive, they perceived that for their families, the situation was often an ongoing source of distress and anxiety. Some participants expressed feelings of guilt for their families' experiences and sometimes felt the situation put a strain on relationships, and this was sometimes overlooked by clinical staff. However, clinical staff acknowledged the strain on relationships and noted that the situation had caused several relationship breakdowns post‐implantation. A couple of participants felt conflicted because they were grateful for the practical support given by family members but also frustrated by their own dependency, particularly if family members were perceived as being overprotective.
Emotional wellbeing
Participants expressed a range of emotions relating to the LVAD. Some described feeling trapped by it, and others described feeling depressed when they first went home but gradually learning to adapt and accept the LVAD. One participant described his experiences every morning when he first got home; he described bursting into tears as he connected to the batteries, but this evolved, and he became more accepting of his situation over time.
Two participants suggested that although they were getting on with the LVAD, they would never fully accept it. Two participants described experiencing flashbacks caused by sudden or loud noises, which resulted in strong emotional reactions that were upsetting and could last several hours. A couple of participants felt that dealing with the emotional impact was harder than the physical aspects of the LVAD.

Abbreviation: LVAD, left ventricular assist device.

**Table 2:** Examples of quotes from life with the LVAD.

Domain	Item concept quotes
Life with the LVAD	
Day‐to‐day life	*I realised that waking up during the night, popping downstairs to make a cup of tea was no longer available to me and I really had to think seriously about just jumping out of bed and things like that because I was tethered to the wall, as it were. (LR08)* *But you work round it like, you know, it is what it is, and you've just got to make the best of it like, yeah, I go shopping, we go to the nursery and have a cup of tea and all that sort of thing, it hasn't restricted me in any way. (LR01)* *You've got to carry it round, you've always got the chance of an alarm going off, you've got to have your spares with you, everything's got to be planned. I suppose in that life that they're leading you can't be spontaneous, everything has to be planned [yeah] and there is always the weight and the luggage that you've constantly got with you. (LR03)* *Being able to cope, their day‐to‐day life, keep having to change, carrying the batteries round, there's a whole … their way of life may have changed. We've got one gentleman who was very active, going to the gym, everything like that and now if feels he can't do that. (STF04)* *So, my routine is, I have a shower only every three days, I would have every day, I go three days, I cover the whole thing up with a waterproof dressing and then after I've had a shower, my waterproof dressing does actually keep the proper dressing dry but just in case. (LR05) … and just feeling that, yes, they've got this device that's ultimately extended their life, but the quality of that life might not be that great either because of the day‐to‐day restrictions …. (STF07)* *So, like obviously they don't have to shower every day, but you can't leave a soggy dressing on so it's either they shower every day, do the dressing every day, or they just have a wash. (STF12)*
The LVAD and me	*I don't think I've properly adjusted to it now because it's only been a year that I've had the LVAD. I'm still finding it hard now. (LR03)* *It took me a while to realise that life could be normal but dealing with all of the bits and pieces wasn't so much of an issue. (LR09)* *You do get your little problems and you get a bit down in certain situations because you can't never be away from batteries or from your lead. But other than that, like I think it's great for considering, you know, two and a half years ago I wouldn't be here. (LR01)* *I would say I am still adjusting now …. Three years, it's still one of those things where you sort of wish you didn't have it but then as I said you've just got to get on with it otherwise you're not here and it's simple things like remember to change your batteries if you're going to go out anywhere, remember to take your spares with you, plug myself into the mains at night. (LR04)* *But it's just the fact that it's there all the time, it's there when you go to bed, it's there when you wake up, wherever you go it goes around with you. And that's one of the hardest things to try and adjust to. (LR04)* *… it depends on the journey people have taken into having an LVAD and for me, I came from declining health, death just around the corner and the LVAD has given me a new lease of life. Now for other people they were perfectly fit, and they've had a heart attack, they went into hospital they came out with an LVAD. So, they're comparing their life before with life afterwards. (LR05)* *I've had people say to me that this wasn't what they signed up for and they didn't realise that it was going to be so difficult. I find you need a very sanguine, phlegmatic and positive attitude … a ‘can do’ sort of attitude. This is not suitable for everyone. (LR08)* *It really depends on the person …. You could have people come in, right after the operation, and they seem quite relaxed, they are getting on with things, they have come to terms with things, they are slowly getting used to and getting into a routine and you can get others that have had them for a couple of years and they're on the waiting list and they can't get rid of it quick enough, they hate it, they really hate it …. (STF05)* *Some have adapted fine, and it becomes part of their routine and part of their life but for others, they really struggle psychologically. They feel depressed and don't want to do anything. (STF04)*
Appearance	*The issue I have is that I have to be selective as to what I'm going to wear when I'm going out. I do miss wearing smart suits and that sort of thing but … hell, I'm alive [laughter]. (LR08)* *Just one thing, if I feel the need to dress up and be smart, that causes some problems. For example, my son is getting married in three weeks' time and I'm not going to look very tidy, but you know, if I looked sort of 40 years younger it might bother me, but it doesn't. (LR01)* *Initially, I was very self‐conscious about it and then I came to the stark realisation that they're the ones with the problem. (LR04)* *When I look at photos of me and the way I looked physically is a bit of a shock because obviously, when you've got the driveline and the batteries, you just look huge. (LR11)* *I remember one woman, she was getting quite upset because of the drive line she was quite feminine and she liked to wear her dresses and what can I do with this drive line and I think, I don't know whether someone was helping her doing some stitching and making it so as the drive line can go through a part of the dress so she could put the dress on, you know, different things like that just to try and help. She just didn't feel feminine, you know, having to carry that about …. (ST04)*
Travel and socialising	*You do lose your confidence in driving, and I never thought I'd say that because I used to drive all over the country. However, when you haven't driven for six months, and somebody has had to drive you around and then to get back in the car …. (LR07)* *On public transport I have to have someone with me …, I get anxious being on my own. So, I basically have to have someone with me all the time. (LR10)* *Emotionally, I think a lot of them have a lot of anxiety You find some people are completely gung‐ho, I had someone phone me and they're literally like, right I'm at the airport, I'm going skiing and I'm like, what, I'm like, ooh and then other people are just petrified of going out the house on their own. So, there is an anxiety level just being on their own or going out and doing things that they want to do that they can't really cope with. (STF12)* *So, if they see the LVAD, you know what will, people generally don't know what they are, or the suspicion about them, and particularly for people who might be of Muslim faith. They really, you know worry that people are going to look and think they've got like a suicide vest on or, and things like that have happened to people where they've been stopped and asked. (STF06)*

Abbreviation: LVAD, left ventricular assist device.

Cognitive interviews were conducted to ensure that items reflected participants' experiences. They also evaluated participants' understanding of the questions and response options to ensure the design and structure of the questionnaire were understood as intended by the authors.^11^, ^22^ Participants for the cognitive interviews were consented separately; one partially sighted participant reviewed the PROM for accessibility for visually impaired participants. The advent of COVID‐19 restrictions meant that cognitive interviews were conducted online. Cognitive interviews took part in two stages; minor modifications were made based on feedback from the first group; these were reviewed by a second group; and no further amendments were required.

### Stage 3 psychometric testing of PROM

Participants were recruited from three UK national heart transplant centres. Potential participants were identified by LVAD co‐ordinators and sent an electronic or hard copy of the patient information sheet, consent form and demographic questionnaire, depending on their preferred method of contact. Recipients returned the forms to the research team, and depending on their preference, they were sent either a link to an online REDCap version of the questionnaire or a pre‐paid hard copy.^23^ Participants were asked if they would be willing to complete a second questionnaire. Participants who agreed were sent a second questionnaire approximately 2 weeks after the first questionnaire was returned to the research team or completed online.

### Methods of psychometric data analysis

Rasch measurement theory was utilised to examine the psychometric properties of the LVAD‐QoL modules.^24^, ^25^ The Rasch model identifies conditions that data should embody if the LVAD‐QoL presents a unidimensional construct and summation of scores can be considered valid. Rasch analysis discloses anomalies in the data and any potential measurement issues.^26^ Data analysis was completed using RUMM2030 software.^27^ Identifying fit to the Rasch model is an iterative process, and individual modules within the LVAD‐QoL were analysed for fit to the model. Data analysis was exploratory, and item fit and dependency were reviewed alongside the conceptual framework, qualitative data and GCM results. Together, these inform decisions on retaining or removing problematic items. Misfitting items were included within other modules if they had face validity, and data from the qualitative interviews and GCM suggested that this was appropriate. The Rasch analysis was repeated, including additional items.

### Tests of fit

Fit to the Rasch model was established using a variety of indicators and fit statistics. Individual tests of fit for each person and item are reflective of the differences between responders' observed and expected responses if the data fit the Rasch model. RUMM2030 automatically clusters responders into equivalent size groups (class intervals) based on their perceived levels of the underlying construct. Several statistics utilise these class intervals, including *χ* ^2^ statistics and residual values. Residual statistics are standardised sums of the deviation of individual scores from expected values summed over all participants. Residual scores approximating ±2.5 indicate an acceptable fit. A significant overall *χ* ^2^ probability suggests that the data do not fit the model, and therefore, further exploration is required.

### Person separation index

The person separation index (PSI) is a reliability index analogous to Cronbach's alpha and demonstrates the extent to which the scale can differentiate between levels of the attribute being measured by the scale, in this case differing levels of quality of life.^26^, ^28^ Like Cronbach's alpha, the PSI is dependent on the number of items within the scale, but the PSI can deal with missing data. A PSI > 0.7 is considered optimal for group delineation.^26^

### Targeting

Due to the small sample size, targeting was the primary aim of the analysis, although sample sizes of 100–150 are thought to ensure stability of person and item calibrations and enable item calibrations on a new scale within a half logit with confidence intervals of between 95% and 99%.^29^, ^30^ Targeting refers to the relative distribution of patient and item locations based on the underlying trait. Patient and item distributions are compared on the same logit scale, where the alignment of the measurement range can be assessed. We reviewed the coverage of item thresholds in the individual modules and the distribution of participants across those modules.^26^, ^29^

### Item independence

The Rasch model requires that responses to items be unrelated. Response dependency occurs when a person's answer to one item is interconnected with their response to another item.^31^ Causes of dependency can include related item content or response options with in‐built dependency.^32^ A classic example would be where a person is affirming an item asking if they can walk 10 m; consequently, they would also affirm an item asking if they can walk 4 m. Residual correlation metrics (<0.2 above the average residual correlation) were used to identify if response dependency was an issue.^31^ Dependency can be dealt with by deleting one or more dependent items post hoc or by creating testlets to absorb the dependency when conceptual groupings are considered legitimate.^32^

### Scoring categories

Rasch analysis examines the scoring categories of items and the extent to which they capture increasing or decreasing levels of the underlying trait. Thresholds are points between adjoining categories where the probability of affirming either category is 50/50. When the affirmation of categories follows a logical hierarchy, thresholds appear ordered. Disordered thresholds may be indicative of redundant scoring categories or poorly worded response options.^26^

### Differential item functioning

Differential item functioning investigates whether responses to individual items by sub‐groups are consistent with their overall perceived levels of health. Here, time points completed and age groups were explored.^26^ Differential item functioning was examined using ANOVA and statistically significant probability.

Once the ‘Rasch factor’ is extracted, residuals should not demonstrate any patterns in the data. Principal component analysis of the residuals and binomial determinants were used to detect if multidimensionality was an issue.^26^ When data fit the Rasch model, recipient and item parameter estimates are positioned on the same log‐odds unit scale as independent parameters. This allows for a linear transformation of the raw scores to be utilised, and estimates of the recipient's quality of life can be measured with confidence.^26^

## Results

### Qualitative interview participants

Eleven recipients took part in the interviews {10 males [90%], median age 64 [interquartile range (IQR) 60–71] years}; participants were experienced in living with an LVAD [median time with LVAD, 25 months (IQR 8–32 months)]. Ten clinical staff completed interviews, including cardiologists, cardiac surgeons, LVAD co‐ordinators, a cardio‐echo physiologist, an auxiliary nurse and a clinical psychologist. Most clinical participants were female (60%) and experienced in working with LVAD recipients [median 48 months (IQR 30–110 months)], with eight (90%) qualified for more than 10 years.

**FIGURE 2:**
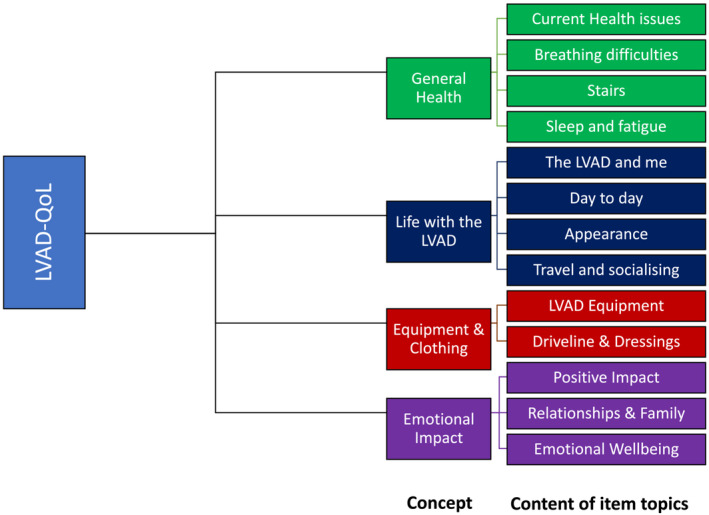
Conceptual framework used in the development of the left ventricular assist device (LVAD) quality of life (LVAD‐QoL). This figure represents the overall conceptual framework, concepts and item topics covered by the LVAD‐QoL patient‐reported outcome measure.

A conceptual framework based on 4 themes and 13 sub‐themes from the qualitative interviews was used to underpin the development of the LVAD‐QoL concepts and modules of items. The four key themes included *general health issues*, *life with the LVAD*, *equipment and clothing* and *emotional impact* (Figure 2).

### Psychometric testing of LVAD‐QoL

#### Participants

Forty‐nine out of 61 (80%) participants completed LVAD‐QoL questionnaires, with 43 (88%) completing them at two time points. Ninety‐two questionnaires in total were completed; 33 participants (67%) completed paper versions of the LVAD‐QoL, and 16 (33%) completed an online version. The median age of participants was 61 (IQR 58–66) years, 42 were men (86%) and most were experienced recipients [median length of time with an LVAD of 33 months (IQR 25–54)].

Initial analysis identified potential issues within the modules (*Table* 3). The total number of misfitting items was low (*n* = 4). Dependency between items was the main concern, and many of the changes to the final LVAD‐QoL modules were instigated because of dependency. Some causes were obvious and related to the structure and content of the questions, which duplicated information, and consequently, redundant items were deleted. For example, two items relating to the driveline (*I am worried about the infection in my driveline*) and (*I worry about getting an infection in the driveline or wound*) had high levels of dependency (0.724); the similarities in item content justified the removal of one item. Another two items, 4 (*I get flashbacks that upset me*) and 7 (*My thoughts keep me awake at night*), were also dependent (0.201). However, information from the qualitative interviews suggested that individually, they were important and sufficiently different to be retained, so a testlet was created to deal with the dependency. None of the items demonstrated differential item functioning for age group or time point.

**Table 3:** Initial Rasch analysis summary fit details.

Domains	Sub‐scales	*N* (missing cases)	Item fit residual	Person fit residual	*χ* ^2^ interaction			PSI	Items		
			Mean (SD)	Mean (SD)	*χ* ^2^	df	*P* (BF c)		Misfitting/no. of items	No. of pairwise dependencies > 0.2 (%)	% Ordered responses
General health	Current health issues	92 (0)	−0.037 (1.048)	0.311 (1.140)	16.886	10	0.077 (0.001)	0.649	0/10	3/45 (7)	50
	Breathing difficulties	85 (7)	0.109 (0.500)	−0.241 (0.749)	6.515	5	0.259 (0.002)	0.389	0/5	0/10 (0)	100
	Stairs	90 (2)	0.256 (2.250)	−0.225 (0.731)	44.058	4	0.000 (0.002)	−0.421	2/4	1/6 (17)	0
	Fatigue and sleep	90 (2)	0.264 (0.491)	−0.270 (1.053)	2.218	6	0.899 (0.002)	0.745	0/6	2/15 (13)	100
Life with an LVAD	Day‐to‐day life	88 (4)	0.092 (0.068)	−0.312 (0.871)	7.210	5	0.206 (0.002)	0.774	0/5	1/10 (10)	80
	LVAD and me	82 (10)	0.200 (1.101)	−0.252 (0.756)	3.398	6	0.757 (0.002)	0.819	0/6	1/15 (7)	63
	Appearance	87 (5)	−0.135 (1.312)	−0.314 (0.972)	12.335	5	0.030 (0.002)	0.746	0/5	1/10 (10)	60
	Travel and socialising	87 (5)	0.117 (1.152)	−0.260 (1.036)	27.645	10	0.002 (0.001)	0.875	1/10	4/45 (9)	40
Equipment and clothing	Equipment and clothing	91 (1)	0.314 (0.879)	−0.163 (1.155)	21.206	12	0.047 (0.0008)	0.748	0/12	11/66 (17)	50
	Driveline and dressings	92 (0)	−0.101 (1.460)	−0.147 (0.938)	33.440	9	0.000 (0.001)	0.767	1/9	5/36 (14)	22
Emotional impact	LVAD positives	79 (13)	−0.620 (2.226)	−0.434 (1.033)	51.422	8	0.000 (0.001)	0.869	1/8	9/28 (32)	50
	Relationships and family life	89 (3)	0.478 (1.136)	−0.092 (0.926)	23.545	8	0.003 (0.001)	0.694	0/8	1/28 (4)	38
	Emotional wellbeing items	91 (1)	−0.080 (1.249)	−0.245 (0.976)	47.016	19	0.000 (0.0005)	0.899	1/19	18/171 (11)	63

Abbreviations: BF c, Bonferroni corrections; LVAD, left ventricular assist device; PSI, person separation index.

The targeting of the range of items to participant locations was good, although slightly skewed as participants were experienced in living with the LVAD (*Table* 4). The ability of the LVAD‐QoL to discriminate between participants' quality of life was assured by the PSI. Once modifications based on initial findings were made, fit to the model was achieved, and unidimensionality was confirmed for the modules (*Table* 4). Combinations of items from different modules resulted in a good fit to the model, ensuring that the content of the final LVAD‐QoL is comprehensive and succinct (*Table* 4). (See also File S1.)

**Table 4:** Final solutions and psychometric properties of the LVAD‐QoL.

Domains	Final questionnaires	Modules and items included	No. of items	No. of cases (missing)	Item fit		Person fit		*χ* ^2^ interaction			Reliability indices		Item characteristics			Unidimensionality test proportions observed		
					Mean	SD	Mean	SD	Value	df	*P*	PSI	% Targeting	% Ordered responses	Misfitting	Pairwise‐dependent items > 0.2	Sig. *t*‐tests	Lower 95% CI	Unidimensionality confirmed
General health	General health questionnaire	General health fatigue, sleep problems	11	92 (0)	0.00	0.981	−1.083	0.821	14.958	11	0.184	0.679	99	55	0	0	0.043	−0.001	Yes
		One item from day‐to‐day life																	
	Breathing difficulties	Breathing and stairs	6	92 (0)	0.00	1.435	−0.453	1.820	5.892	5	0.317	0.810	97	80	0	0	0.045	0.000	Yes
Life with the LVAD	The LVAD and me	Day‐to‐day life, LVAD and me	10	92 (0)	0.00	0.969	−0.503	1.152	12.781	10	0.236	0.821	100	60	0	0	0.043	−0.001	Yes
		Appearance																	
	Travel and socialising	Travel and socialising	8	92 (0)	0.00	0.797	−0.210	1.452	10.016	8	0.264	0.837	92	38	0	0	0.058	0.012	Yes
	LVAD equipment and driveline	Equipment; driveline and dressings	11	92 (0)	0.00	1.566	−0.940	0.747	11.177	11	0.429	0.701	97	46	0	0	0.076	0.032	Yes
Emotional impact	LVAD positives	LVAD positives	5	90 (2)	0.00	0.302	0.387	2.420	7.710	5	0.173	0.841	73	100	0	0	0.045	−0.007	Yes
	Family and relationships	Family and relationships	7	90 (2)	0.00	0.826	−0.002	1.027	9.558	7	0.215	0.713	96	43	0	0	0.034	−0.011	Yes
	Emotional wellbeing	Emotional wellbeing	10^a^	92 (0)	0.00	0.950	−1.162	1.422	16.568	10	0.084	0.854	96	90	0	0	0.056	0.011	Yes

Abbreviations: CI, confidence interval; LVAD, left ventricular assist device; LVAD‐QoL, LVAD quality of life; PSI, person separation index.

^a^ Includes one testlet item consisting of Items 4 and 7 as part of the final solution for dependency.

## Discussion

Using a mixed‐methods approach with the involvement of LVAD recipients has identified the key issues that are important to LVAD recipients within the UK, and this has underpinned the development of the LVAD‐QoL for use in future clinical practice and research. The GCM and qualitative interviews identified key issues encountered by LVAD recipients during their recovery journey and supported the development of a conceptual framework and the LVAD‐QoL. The results of the psychometric analysis identified a few misfitting items, confirming that the content and face validity of the LVAD‐QoL were reflective of the recipients' experiences. Rasch analysis further tested the relative importance of the items and evaluated the structure and content of the different modules, allowing further refinement of the items and the delivery of a succinct measurement tool.

Dependency was found between some items, and when reviewed, it was clear that some items were conceptually similar and therefore duplicating information. Removing duplicate items dealt with this type of dependency. However, there were a few cases where a solution was not so obvious. Two items were considered conceptually different because one item referred to flashbacks during the day and the other item referenced thoughts keeping participants awake at night. Discussions within the qualitative interviews suggested that some participants had experienced post‐traumatic experiences linked to their stay in intensive care, and sudden or loud noises could provoke an emotional response. While this may have been linked to their sleeping difficulties, it was thought that these were conceptually different. To deal with this dependency, a testlet was created in the analysis.

Most of the item thresholds were ordered, although potential disordered thresholds should be revisited with a larger sample size. The targeting of the items to participants was good, as shown by the person/item locations and the percentages of participant locations captured by the items within the LVAD‐QoL modules. This is important as participants were generally experienced in living with the LVAD, and this could have skewed results. Further data collection following a recipient's recovery journey will be needed to confirm the sensitivity of the LVAD‐QoL in capturing change scores over time. The time and care invested in developing items for the LVAD‐QoL were supported by the results, as demonstrated by the low number of misfitting items. Rasch analysis confirmed the content validity of the LVAD‐QoL.

Throughout this project, LVAD recipient experiences have been central to its conception and design. Our patient partner group has been active in their support, including the development of the original grant applications, patient‐facing documents and ethics applications. They have given us valuable feedback on the structure and content of the draft LVAD‐QoL as well as reviewing the results of the psychometric analysis.

One issue for the study was the recruitment of female participants. Fewer women are implanted with an LVAD and often present later and with poorer health outcomes. Our recruitment numbers may be reflective of this, as some of the women recruited early on in the study were transplanted before they could take part in an interview or complete the LVAD‐QoL.^33^ Consequently, there may have been additional areas that were problematic for women that were not raised in the qualitative interviews. We anticipated that the appearance and weight of the equipment might be more of an issue for females, but this was raised by both male and female participants in the GCM and the qualitative interviews. Participants completing the LVAD‐QoL were also encouraged to provide additional information if they thought anything was missing from the questionnaire; no additional issues were raised by any participants.

Several COVID‐19‐related issues impacted the study. Originally, it had been planned to give participants the option of face‐to‐face interviews, but these had to be moved online or conducted as telephone interviews. The advantage of this was that we were able to interview participants from a large, geographically dispersed group. Staff interviews were originally planned as focus groups, but many of the clinicians were moved to frontline duties. To accommodate these restrictions, we conducted individual online interviews with a wide range of staff. Implementing these changes required additional ethical approvals, and the prioritisation of COVID‐19‐related studies consequently delayed our research. These delays meant that some of the earlier participants had died or been transplanted before the LVAD‐QoL was ready for testing. Some participants, especially in later interviews, discussed the impact of COVID‐19 on their levels of anxiety, especially in relation to travel and going out, and this may have influenced responses in the LVAD‐QoL. Changes in clinical practice and COVID‐19‐related issues meant that fewer patients were implanted, and therefore, the numbers of potential participants were lower than initially expected. This impacted the number of recipients available for psychometric testing of the LVAD‐QoL.

Transplant centre staff work hard to ensure that potential recipients understand the implications of receiving the LVAD. However, it may not fully prepare recipients for the reality of the long‐term consequences of implantation. The structure of health care in the UK and the dearth of available transplants result in LVAD implants becoming a destination therapy by default. Consequently, recipients may live with the LVAD for an extended period. As one consultant commented, ‘I know they're bridged to transplant but often that's not a short bridge.’ Monitoring a recipient's long‐term recovery requires input from a highly skilled clinical team. Previous studies have shown that the post‐implant journey is not without complications, and while welcoming relief from previous symptoms, life for recipients does not necessarily return to ‘normal’.^6^

Therefore, the development of the LVAD‐QoL is a useful adjunct for monitoring the post‐implant journey and could be used to identify recipients who are struggling, as well as inform care pathways and resource management. Additional testing of the LVAD‐QoL is needed to ensure that the items are sensitive enough to capture changes over time, the stability of the item locations and cultural relevance for wider groups with different populations. As identified in the LVAD‐QoL, the support of family and carers was important for participants, but this can also impact their quality of life. Future development of a caregiver burden questionnaire is also needed to ensure that this can be part of ongoing clinical care in the future. Monitoring recipients in future research or design evolutions may help identify adverse events, key milestones or issues that, if not addressed, could be detrimental to a recipient's physical and psychological health.

Future research will need to evaluate the sensitivity of the LVAD‐QoL in identifying recipients requiring clinical interventions as well as capturing changes in the post‐implant journey over time. Confirmation of the psychometric validity of the scale with larger clinically defined groups is also recommended.

## Conclusions

The LVAD‐QoL has been developed using a robust methodology incorporating modern psychometrics and input from LVAD recipients throughout its development, confirming the content validity and importance of items to LVAD recipients. The LVAD‐QoL will be a useful adjunct for capturing recipients' post‐implant journey and a useful tool for evaluating future design evolutions, clinical practice and research.

## Funding

This work was supported by funding from the British Heart Foundation (Ref. No. PG/18/58/33944). The study funders did not have any role in the study design, the collection, analysis and interpretation of the data, the writing of the report or the decision to submit the article for publication.

## Conflict of interest statement

ALS was supported by funding from the British Heart Foundation; in addition, she received funding from the National Institute for Health Research Invention for Innovation, Medical Research Council, National Institute for Health Research MedTech and In Vitro Diagnostics Co‐operative‐Trauma Management, National Institute for Health Research Clinical Research Network, the National Institute for Health Research Birmingham Biomedical Research Centre, and the National Institute for Health Research Surgical Reconstruction and Microbiology Research Centre at the University of Birmingham and University Hospitals Birmingham National Health Service Foundation Trust outside the submitted work.

CM receives funding from the National Institute for Health Research Surgical Reconstruction and Microbiology Research Centre, the NIHR BTRU in Precision Transplant and Cellular Therapeutics, Innovate UK and Anthony Nolan and has received personal fees from Aparito outside the submitted work.

MJC is a NIHR Senior Investigator and receives funding from the NIHR Birmingham Biomedical Research Centre, NIHR Surgical Reconstruction and Microbiology Research Centre, NIHR Birmingham‐Oxford Blood and Transplant Research Unit (BTRU) in Precision Transplant and Cellular Therapeutics and NIHR ARC West Midlands at the University of Birmingham and University Hospitals Birmingham NHS Foundation Trust, Health Data Research UK, Innovate UK (part of UK Research and Innovation), Macmillan Cancer Support, European Regional Development Fund—Demand Hub, SPINE UK, UKRI, UCB Pharma, GSK and Gilead Sciences. MJC receives personal fees from Astellas, Aparito Ltd, CIS Oncology, Takeda, Merck, Daiichi Sankyo, Glaukos, GSK, ICON and the Patient‐Centered Outcomes Research Institute (PCORI) outside the submitted work. In addition, a family member owns shares in GSK.

DAL has received investigator‐initiated educational grants from Bristol‐Myers Squibb (BMS) and Pfizer, has been a speaker for Bayer, Boehringer Ingelheim and BMS/Pfizer and has consulted for BMS and Boehringer Ingelheim, all outside the submitted work.

The views expressed in this article are those of the author(s) and not necessarily those of BHF, the National Institute for Health Research or the Department of Health and Social Care.

## Supporting information


**Data S1.** Supporting Information.

## Data Availability

Anonymised data can be accessed upon request from the Centre for Patient Reported Outcomes Research, University of Birmingham.
