# Supplementary material for: Development of a quality of life measure for left ventricular assist device recipients using a mixed methods approach
Source: ESC Heart Fail. 2024 Jun 14;11(5):3167–79. doi: 10.1002/ehf2.14850 (PMC11424331; doi:10.1002/ehf2.14850)
Supplement: Supplementary file 1 — Data S1. Supporting Information. [file EHF2-11-3167-s001.pptx]

## Slide 1
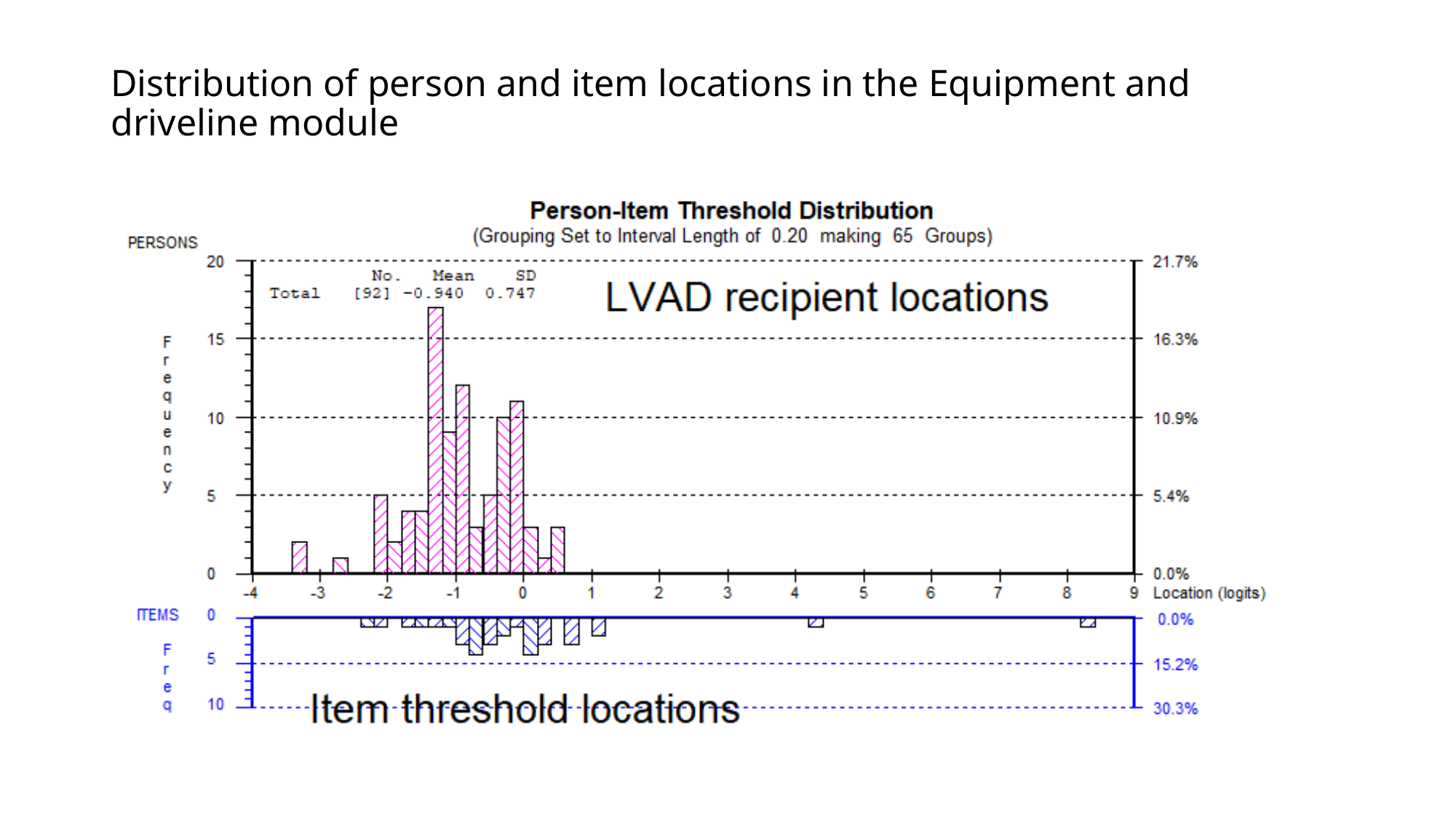

# Distribution of person and item locations in the Equipment and driveline module

## Slide 2
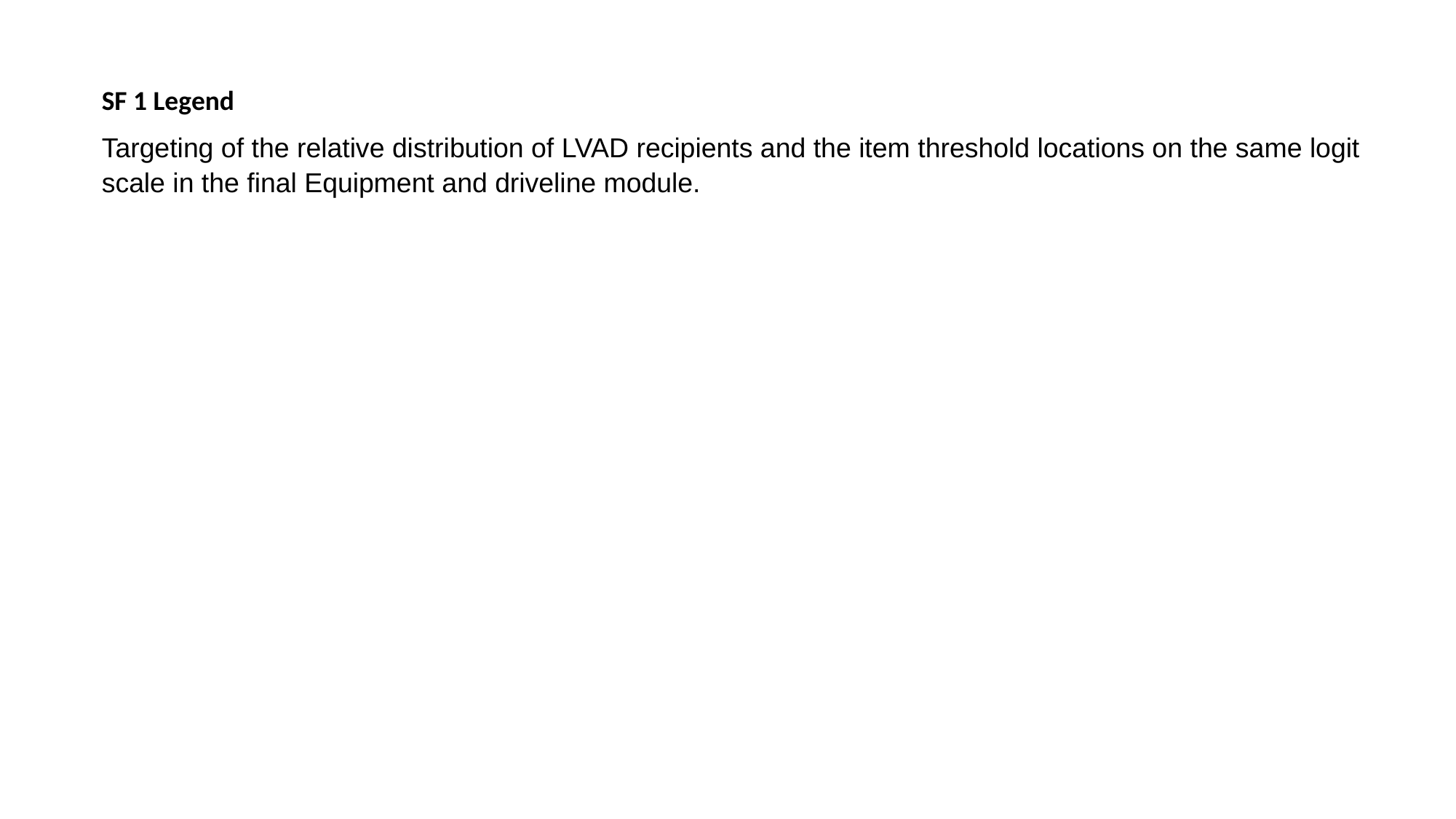

SF 1 Legend
Targeting of the relative distribution of LVAD recipients and the item threshold locations on the same logit scale in the final Equipment and driveline module.
